# Geochemical data of Al-rich diopside pyroxenites from the Premosello mantle peridotite massif, Ivrea-Verbano Zone, Southern Alps

**DOI:** 10.1016/j.dib.2025.111362

**Published:** 2025-02-03

**Authors:** Abimbola C. Ogunyele, Alessio Sanfilippo, Mattia Bonazzi, Maria C. Lopez Suarez, Alberto Zanetti

**Affiliations:** a Department of Earth and Environmental Sciences, University of Pavia, Via Ferrata 1, 27100 Pavia, Italy; b CNR – Institute of Geosciences and Earth Resources, Via Ferrata 1, 27100 Pavia, Italy; c CNR – Institute of Geosciences and Earth Resources, Via Moruzzi 1, 56124 Pisa, Italy

**Keywords:** Orogenic peridotite massif, Mantle pyroxenite, Mineral geochemistry, Ivrea-Verbano Zone, Continental lithosphere, Mohorovičić discontinuity

## Abstract

Pyroxenites of different generations and composition are usually found within orogenic peridotite massifs and in mantle xenoliths entrained in volcanic rocks. Orogenic peridotite massifs, however, offer great advantages over xenolith studies because structural relationships which formed in the mantle before exhumation are often preserved, and crosscutting relationships between dykes of different generations and composition can be readily observed. Numerous orogenic peridotite massifs occur in the Ivrea-Verbano Zone (IVZ) in the western Southern Alps, providing petrologists, geochemists and geophysicists a natural laboratory to study and understand Earth's mantle processes and evolution. We here report new geochemical data for peculiar Al-rich diopside pyroxenites which crosscut the Premosello mantle peridotite massif in central IVZ close to the transition to the continental crust (i.e., the Moho region). The pyroxenite is composed of Al-rich clinopyroxene (Cpx), spinel (Sp), and amphibole (Amph), with subordinate amounts of olivine (Ol), and occasional orthopyroxene (Opx) as an accessory phase. Electron microprobe (EMP) and laser-ablation inductively coupled plasma mass spectrometry (LA-ICP-MS) analysis were performed to measure the major and trace elements contents of the mineral phases of the pyroxenite. Major element composition of each mineral phase is characterized by uniform Mg# (Cpx: 0.88-0.90; Ol: 0.87-0.88; Sp: 0.73-0.77; Amph: 0.84-0.87; Opx: 0.87) and high Al_2_O_3_ contents (except in Ol). The trace element composition of Cpx and Amph from the Al-rich diopside pyroxenite shows strong rare earth elements (REE) fractionation and enrichments in LREEs and MREEs over the HREEs, and is distinct from other pyroxenite compositions (i.e., Al-augite and Cr-diopside pyroxenites) reported from the Premosello and other IVZ lherzolitic peridotite massifs. The geochemical data presented herein, therefore, offer valuable insights into the compositional variability and formation processes of mantle pyroxenites, and may contribute to unravelling the broader evolutionary history of the Earth's subcontinental lithospheric mantle, in particular at Moho levels.

Specifications Table

**Table:** 

Subject	Geochemistry and Petrology
Specific subject area	Major and trace elements geochemical data of mineral phases from Al-rich diopside pyroxenites from Premosello mantle peridotite massif, central Ivrea-Verbano Zone
Type of data	Table, Figure
Data collection	Dark-coloured pyroxenites exhibiting reactive boundaries and crosscutting relationships with the host Premosello peridotite were investigated. Three representative samples of the pyroxenites were selected for petrographic and mineral major and trace elements analysis. Petrographic observations were made on thin sections using optical microscope. Major and trace elements contents of mineral phases were determined in situ in thin sections by electron microprobe (EMP) and laser-ablation inductively coupled plasma mass spectrometry (LA-ICP-MS).
Data source location	Data are stored at the CNR – Institute of Geosciences and Earth Resources, Pavia (Italy) Sample location: Premosello Chiovenda, Val d'Ossola, Italy GPS (WGS84) coordinates: 8° 19′ 12′’ E, 46° 00′ 19′’ N
Data accessibility	Repository name: Mendeley Data Data identification number: 10.17632/nsytmc8j2p.1 Direct URL to data: https://data.mendeley.com/datasets/nsytmc8j2p/1
Related research article	None

## 1 Value of the Data

• The presented data, which is also accessible in [1], represents the first-ever geochemical data reported for Al-rich diopside pyroxenites crosscutting the Premosello peridotite massif in central IVZ.
• Currently, only a few geochemical datasets are available in the literature [2] for a compositionally different type of pyroxenite (i.e., Al-augite pyroxenite) in the Premosello massif.
• The presented data therefore offer valuable insights into the compositional variability and formation processes of pyroxenitic lithologies that occur in the upper mantle.
• The data may be a precious resource for better defining the composition of the upper mantle, the pyroxenite-peridotite interaction processes as well as using it to better understand the role of the pyroxenites in controlling the chemical variability of mantle-derived melts.
• The data may also contribute to understanding the broader evolutionary history of the Earth's subcontinental lithospheric mantle, in particular, in sections close to the continental crust-mantle boundary.

## 2 Background

The continental lithosphere preserves much of Earth's history, and its rigidity plays a crucial role in plate tectonics. As such, studying the petrology, chemical composition, and physical properties of the continental lithosphere is essential for understanding the Earth's composition, formation and evolutionary processes [3]. While the continental crust is accessible in many regions, the lithospheric mantle remains largely out of reach. Although geophysical techniques, such as seismology, can probe the deep mantle, field-based petrologic and geochemical studies are typically limited to specific sites. These include areas where mantle xenoliths are brought to the surface by volcanic rocks like alkali basalts and kimberlites, as well as orogenic peridotite massifs. The Ivrea-Verbano Zone (IVZ) in the western Southern Alps is one of such unique places in the world where fragments of the subcontinental lithospheric mantle outcrops on the surface, the result of tectonic exhumation of the upper mantle to lower crustal levels and subsequent tilting during the Alpine orogeny [4].

**Fig. 1:**
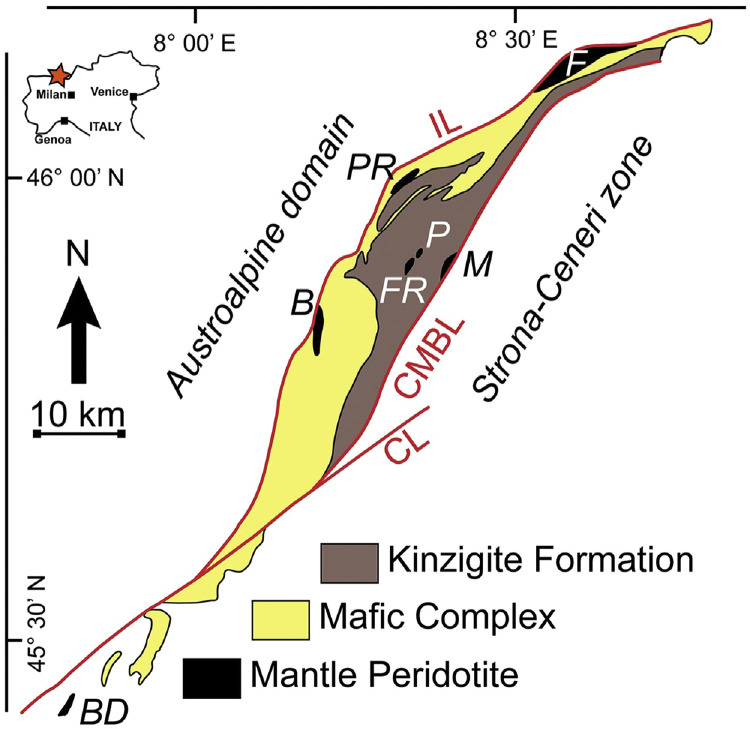
Geological sketch map of the Ivrea-Verbano Zone showing the location of the Premosello (PR) and other major mantle peridotite massifs (F: Finero, P: Alpe Piumero, M: Alpe Morello, FR: Alpe Francesca, B: Balmuccia, BD: Baldissero). IL: Insubric Line; CL: Cremosina Line; CMBL: Cossato-Mergozzo-Brissago Line (modified after [5]).

**Fig. 2:**
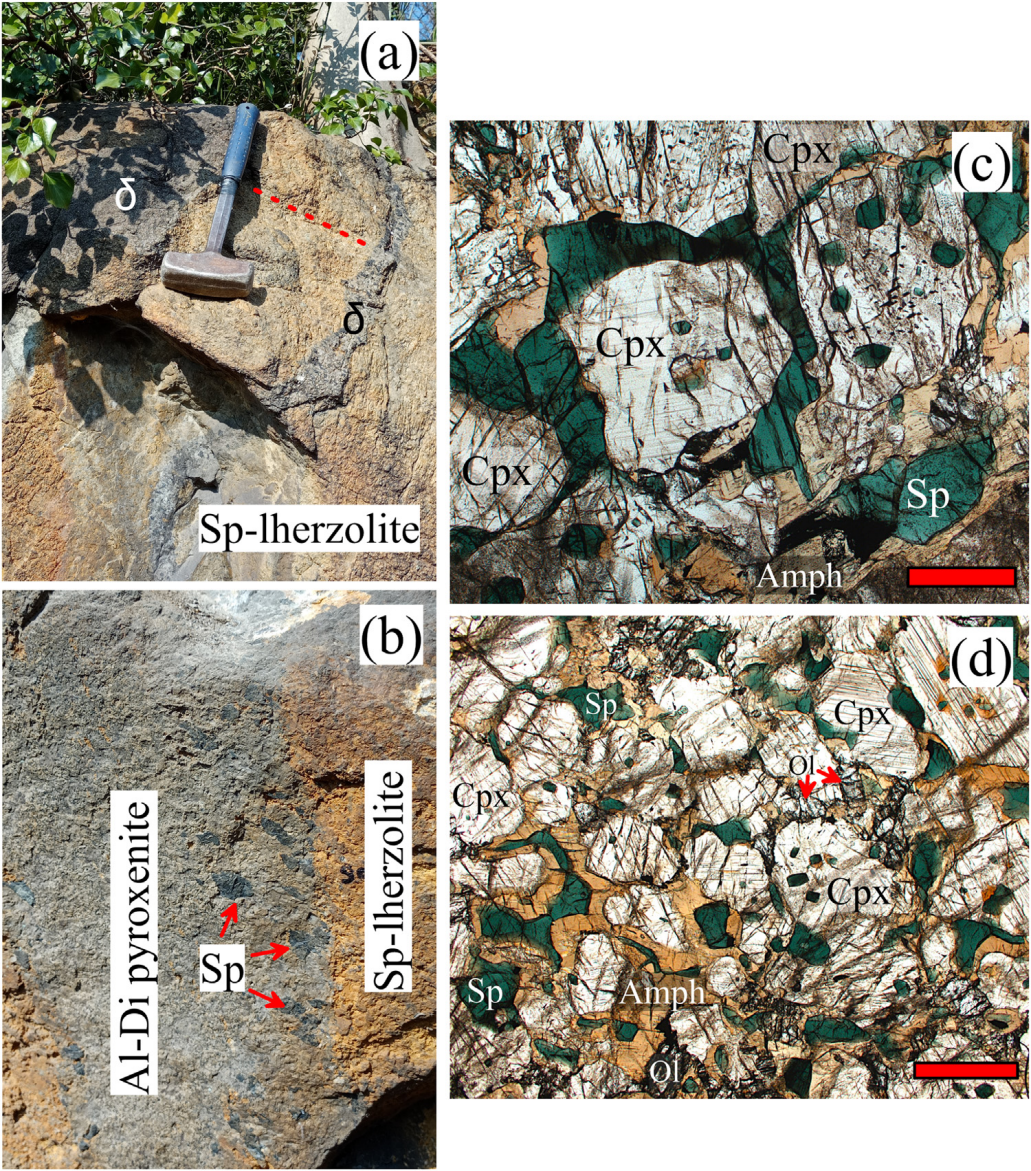
Field (a,b) and petrographic (c,d) features of the Premosello Al-rich diopside pyroxenites. (a) Photograph of the Premosello spinel lherzolite (peridotite) crosscut by dykes of Al-rich diopside pyroxenites (δ) (red broken line indicates the strike direction of the mantle foliation); (b) close-up view of the Premosello Al-rich diopside pyroxenites exhibiting sharp boundaries with the host peridotite and alignment of spinel megacrysts along the boundaries (width of dyke is about 10 cm); (c,d) photomicrographs (in plane polarized light) of the studied pyroxenite showing large clinopyroxene crystals (oikocrysts) enclosing numerous smaller crystals of green spinel and brown amphibole (chadacrysts). Spinel and amphibole also occur within interstices of clinopyroxene, sometimes forming rims around the latter. Red bars in (c,d) represent 1 mm. Cpx – clinopyroxene; Sp – spinel; Amph – amphibole; Ol – olivine.

In this contribution, we present new geochemical data for peculiar Al-rich diopside pyroxenite dykes that crosscut the Premosello peridotite massif in central IVZ (Figs. 1 and 2). These data offer valuable insights into the compositional variability and formation processes of mantle pyroxenites, and may contribute to unravelling the broader evolutionary history of the Earth's subcontinental lithospheric mantle, in particular, in sections close to the continental Moho.

## 3 Data Description

### 3.1 The Premosello peridotite massif: geological context and petrology

The Premosello peridotite massif is one of tens of orogenic peridotite massifs outcropping very close and broadly parallel to the Insubric Line in the IVZ, western Southern Alps (Fig. 1) [2,6]. Other prominent, kilometer-scale peridotite massifs are located at Balmuccia and Baldissero in southern IVZ, as well as at Finero in northern IVZ (see Fig. 1). The central and southern “Balmuccia-type” massifs comprising Premosello, Balmuccia and Baldissero massifs are predominantly lherzolitic differing in composition, degree of depletion and metasomatic overprint from the northern “Finero-type” massif, which is pervasively metasomatized, and harzburgitic to dunitic in character [4]. Due to the largely unserpentinized nature, lithological variability (characterized by harzburgite, dunite, lherzolite, pyroxenite, chomitite, etc) and lack of significant low-temperature alteration of these massifs, they have been the subject of numerous geochemical, structural and geophysical studies to unravel the nature, chemical composition, rheology and evolutionary processes of the Earth's continental lithospheric mantle [e.g., [2], [3], [4], [5], [6], [7], [8], [9], [10], [11], [12], [13], [14]].

The Premosello mantle peridotite is predominantly composed of porphyroclastic, spinel-facies clinopyroxene-poor (∼5 vol. % Cpx) lherzolites, intruded by numerous pyroxenites of varying generations and compositions. The latter includes Cr-diopside pyroxenite parallel to the mantle foliation and discordant websterite, Al-augite pyroxenite, Al-diopside pyroxenite, gabbroic dykes, and spinel-amphibole-rich layers (Fig. 2a and b) [2,6]. The mantle peridotite shows magmatic contact with an overlying layered intrusive crustal body mainly formed by amphibole-rich gabbros with subordinate pyroxenites, documenting a typical petrographic Mohorovičić discontinuity. The petrochemistry and geochronology of such a gabbroic body are so far poorly constrained. It is speculatively considered part of the trans-crustal Upper-Carboniferous – Lower Permian igneous Mafic Complex of the IVZ developed by underplating magmatism which affected the Variscan-age lower crustal granulite-facies metavolcano-sedimentary sequence (i.e., the Kinzigite Formation, see Fig. 1).

Recent geochemical and Nd-Hf isotopic study [6] of the Premosello lherzolite, together with the Balmuccia and Baldissero lherzolites, indicate that these lherzolites are fragments of young, fertile subcontinental lithospheric mantle (SCLM) that were accreted during the Devonian period (ca. 370 Ma). Significant geochemical and isotopic data also exist for the different generations and compositions of pyroxenites in the Balmuccia lherzolite massif [e.g., 3,[7], [8], [9],^15^], however, geochemical data on pyroxenites from the Premosello [e.g., 2] massif are limited. Consequently, a complete picture of the evolutionary history of the SCLM beneath the IVZ is still lacking.

### 3.2 Al-rich diopside pyroxenite

#### 3.2.1 Petrography and texture

The studied pyroxenite is primarily composed of Al-rich clinopyroxene (∼70 vol. %), spinel (∼10 vol. %), and amphibole (∼15 vol. %), with subordinate amounts of olivine (∼4 vol. %), and occasional orthopyroxene (∼1 vol. %) as an accessory phase (Fig. 2). This rock is dark-coloured, with widths of up to ∼15 cm, and exhibits sharp but irregular and reactive boundaries, and crosscutting relationships with the host peridotite (Fig. 2a and b). Spinel megacrysts are typically aligned along the pyroxenite's margins (Fig. 2b). The pyroxenite is characterized by medium- to coarse-grained, poikilitic textures where large, euhedral to subhedral clinopyroxene crystals (oikocrysts) enclose numerous smaller crystals of green spinel and brown amphibole (chadacrysts) (Fig. 2c and d). Large clinopyroxene crystals are also usually surrounded by spinel and amphibole. Olivine, and rare orthopyroxene, occurs within mineral interstices and are sometimes surrounded by amphibole (Fig. 2d).

#### 3.2.2 Mineral major and trace elements geochemistry

**Fig. 3:**
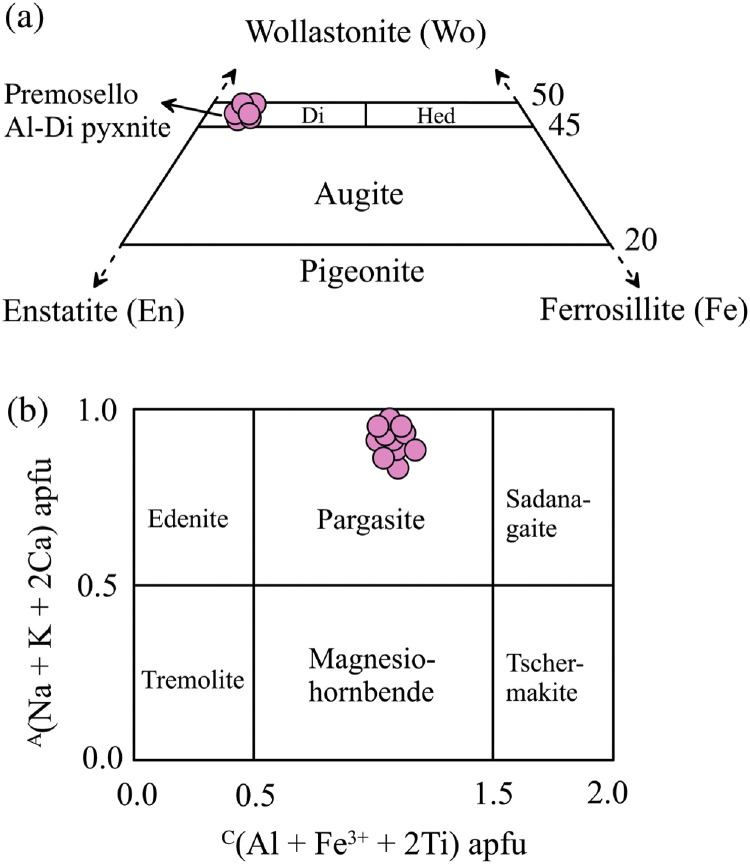
Classification of (a) clinopyroxene and (b) amphibole in the Premosello Al-rich diopside pyroxenite. The clinopyroxene and amphibole classifications are according to [16] and [17]. Di – diopside; Hed – hedenbergite.

**Fig. 4:**
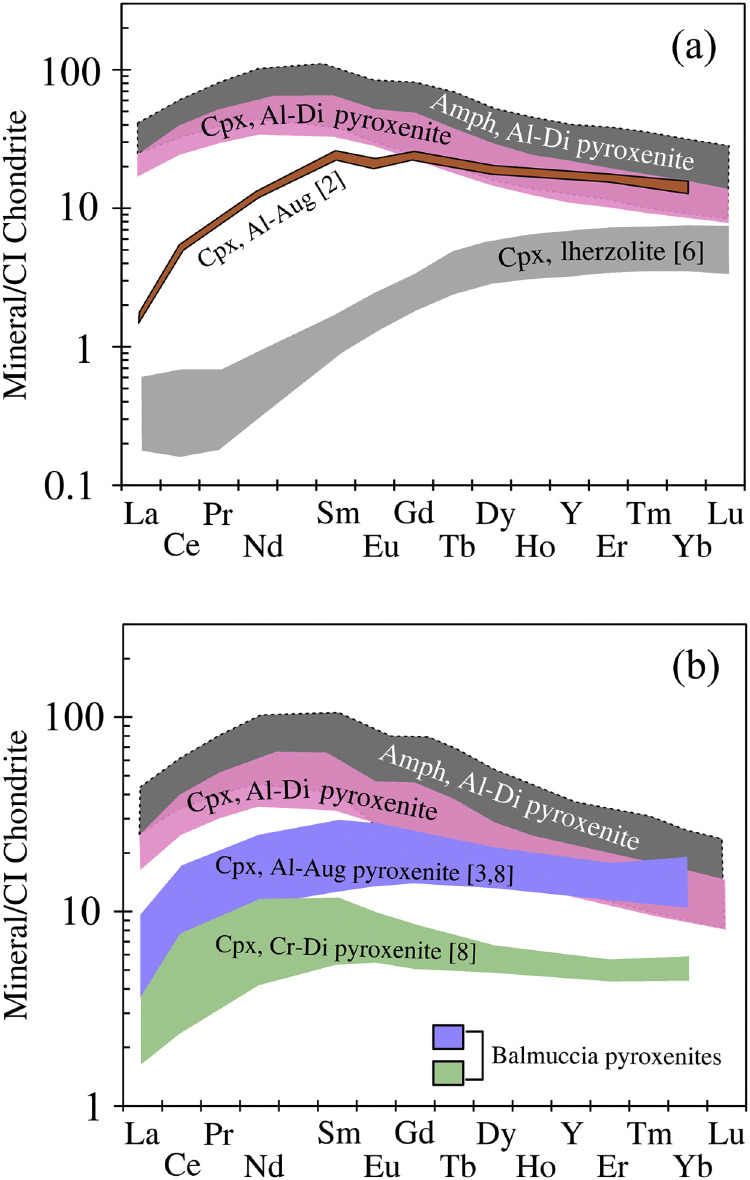
CI Chondrite-normalized REE patterns of clinopyroxene (Cpx) and amphibole (Amph) from the Premosello Al-rich diopside (Al-Di) pyroxenite compared with (a) Cpx from Al-augite (Al-Aug) pyroxenite [2] and spinel lherzolite [6] from the Premosello peridotite massif, and (b) Cpx from Cr-diopside (Cr-Di) [8] and Al-augite (Al-Aug) pyroxenites [3,8] from the Balmuccia peridotite massif (Fig. 4b). CI Chondrite values are from [18].

Major and trace elements analysis were performed on individual grains of mineral phases (clinopyroxene, olivine, spinel, amphibole and orthopyroxene) from three representative samples (PR3-1, PR3-2 and PR3-3) of the Premosello Al-rich diopside pyroxenite. The mineral major and trace element datasets are presented in Table 1, Table 2, Table 3, Table 4, Table 5, Table 6, Table 7, Table 8, Figs. 3 and 4, and in Ogunyele et al. [1], respectively. Clinopyroxene exhibits uniform Mg# (0.88-0.90), low Cr_2_O_3_ (≤ 0.11 wt. %), and high Al_2_O_3_ (4.92-7.17 wt. %), CaO (20.63-23.16 wt. %) and wollastonite contents (Wo: 44.7-50.7), and is classified as diopside (according to [16]; Fig. 3a). Olivine shows magnesian forsterite content (Fo) from 0.87 to 0.88. Spinel is enriched in Al_2_O_3_ (62.89-67.01 wt. %), depleted in Cr_2_O_3_ (0.27-0.90 wt. %; Cr# < 0.01) and show Mg# from 0.73 to 0.77. Amphibole is pargasitic (according to [17]; Fig. 3b) with Mg# from 0.84 to 0.87 and Al_2_O_3_ from 13.63 to 16.06 wt. %. Orthopyroxene is predominantly enstatite (En_86_Fe_14_) with Mg# of ∼0.87 and Al_2_O_3_ content of 4.08 wt. %.

**Table 1:** Major elements composition (in wt. %) and atoms per formula unit of clinopyroxene (crystal 1-13) and orthopyroxene (crystal 53) from Premosello Al-rich diopside pyroxenites.

Sample ID	PR3-1	PR3-1	PR3-1	PR3-1	PR3-1	PR3-2	PR3-2	PR3-2	PR3-2	PR3-3	PR3-3	PR3-3	PR3-3	PR3-2
Crystal ID	1	2	3	4	5	6	7	8	9	10	11	12	13	53
Comments	Core	Core	Core	Core	Core	Core	Core	Core	Core	Core	Core	Core	Core	Core
SiO_2_	51.76	51.51	51.31	51.69	50.47	51.32	50.15	51.36	51.43	50.87	50.74	51.06	50.91	55.36
TiO_2_	0.77	0.90	0.93	0.75	0.99	0.88	1.26	0.99	0.64	1.18	1.08	1.04	1.20	0.23
Al_2_O_3_	6.49	6.41	6.23	5.40	6.73	4.92	7.17	6.04	6.25	6.79	6.53	6.73	6.49	4.08
Cr_2_O_3_	0.01	0.02	0.11	0.04	0.02	0.05	0.02	0.07	0.00	0.00	0.00	0.04	0.07	0.03
FeO_T_	2.97	2.95	3.08	3.53	3.91	3.38	3.26	3.16	3.13	3.09	3.07	3.04	3.23	8.86
MnO	0.07	0.07	0.11	0.10	0.10	0.14	0.12	0.12	0.13	0.09	0.10	0.07	0.07	0.16
NiO	0.00	0.04	0.01	0.03	0.02	0.03	0.00	0.00	0.10	0.02	0.04	0.02	0.00	0.05
MgO	14.33	14.38	14.46	14.98	16.11	14.98	14.47	14.78	14.76	14.66	14.36	14.31	14.24	31.92
CaO	22.46	22.92	22.63	22.34	20.63	23.16	22.97	22.67	22.49	22.30	23.08	22.92	22.74	0.41
Na_2_O	1.41	1.11	1.10	1.13	0.71	0.74	0.88	1.26	1.37	1.30	1.17	1.30	1.30	0.00
K_2_O	0.01	0.00	0.00	0.02	0.00	0.01	0.00	0.01	0.00	0.01	0.00	0.00	0.00	0.00
P_2_O_5_	0.16	0.13	0.16	0.15	0.13	0.17	0.13	0.11	0.16	0.14	0.16	0.16	0.15	0.00
Total	100.45	100.44	100.14	100.15	99.82	99.78	100.43	100.57	100.47	100.45	100.33	100.69	100.39	101.10
Normalization to 6 oxygens and 4 cations per formula unit														
Si	1.870	1.865	1.864	1.876	1.834	1.876	1.819	1.854	1.856	1.837	1.839	1.842	1.845	1.909
Ti	0.021	0.025	0.025	0.020	0.027	0.024	0.034	0.027	0.017	0.032	0.029	0.028	0.033	0.006
Al	0.276	0.274	0.267	0.231	0.288	0.212	0.307	0.257	0.266	0.289	0.279	0.286	0.277	0.166
Cr	0.000	0.001	0.003	0.001	0.001	0.001	0.001	0.002	0.000	0.000	0.000	0.001	0.002	0.001
Fe^2+^	0.048	0.064	0.063	0.053	0.078	0.064	0.051	0.027	0.011	0.028	0.027	0.027	0.041	0.253
Fe^3+^	0.041	0.025	0.030	0.055	0.041	0.039	0.048	0.068	0.084	0.065	0.066	0.065	0.057	0.003
Mn	0.002	0.002	0.004	0.003	0.003	0.004	0.004	0.004	0.004	0.003	0.003	0.002	0.002	0.005
Ni	0.000	0.001	0.000	0.001	0.001	0.001	0.000	0.000	0.003	0.001	0.001	0.001	0.000	0.001
Mg	0.772	0.776	0.783	0.811	0.873	0.816	0.782	0.795	0.794	0.789	0.776	0.770	0.769	1.641
Ca	0.869	0.889	0.881	0.869	0.803	0.907	0.893	0.877	0.870	0.863	0.896	0.886	0.883	0.015
Na	0.099	0.078	0.078	0.079	0.050	0.053	0.062	0.088	0.096	0.091	0.082	0.091	0.091	0.000
Cations	4.000	4.000	4.000	4.000	4.000	4.000	4.000	4.000	4.000	4.000	4.000	4.000	4.000	4.000
Mg#	0.90	0.90	0.89	0.88	0.88	0.89	0.89	0.89	0.89	0.89	0.89	0.89	0.89	0.87
Wo	50.2	50.6	50.0	48.5	44.7	49.5	50.2	49.5	49.4	49.4	50.7	50.6	50.4	0.8
En	44.5	44.2	44.5	45.3	48.5	44.6	44.0	44.9	45.1	45.1	43.9	44.0	43.9	85.6
Fe	5.3	5.2	5.5	6.2	6.8	5.9	5.8	5.6	5.6	5.5	5.4	5.4	5.7	13.6

**Table 2:** Major elements composition (in wt. %) and atoms per formula unit of olivine from Premosello Al-rich diopside pyroxenites.

Sample ID	PR3-1	PR3-1	PR3-1	PR3-1	PR3-2	PR3-2	PR3-2	PR3-3	PR3-3	PR3-3	PR3-3
Crystal ID	14	15	16	17	18	19	20	21	22	23	24
Comments	Core	Core	Core	Core	Core	Core	Core	Core	Core	Core	Core
SiO_2_	40.57	39.30	40.38	40.33	40.39	40.39	40.56	40.11	40.40	40.76	40.16
TiO_2_	0.01	0.01	0.00	0.00	0.00	0.01	0.01	0.05	0.01	0.00	0.02
Al_2_O_3_	0.00	0.01	0.00	0.00	0.01	0.01	0.00	0.02	0.00	0.01	0.01
Cr_2_O_3_	0.00	0.03	0.00	0.03	0.02	0.00	0.04	0.00	0.06	0.02	0.00
FeO_T_	12.67	12.82	12.25	11.61	12.15	11.74	12.27	11.91	11.92	12.29	11.81
MnO	0.21	0.24	0.20	0.23	0.24	0.19	0.17	0.25	0.25	0.25	0.28
NiO	0.19	0.05	0.17	0.21	0.12	0.14	0.18	0.13	0.17	0.13	0.17
MgO	47.48	46.67	47.86	49.06	48.35	48.88	48.44	48.64	48.77	48.30	48.00
CaO	0.01	0.29	0.02	0.01	0.01	0.00	0.02	0.02	0.03	0.00	0.02
Na_2_O	0.00	0.03	0.00	0.02	0.00	0.02	0.00	0.02	0.00	0.00	0.00
K_2_O	0.00	0.00	0.01	0.01	0.02	0.00	0.01	0.00	0.00	0.00	0.00
Total	101.13	99.46	100.89	101.50	101.31	101.38	101.69	101.16	101.61	101.76	100.47
Normalization to 4 oxygens and 3 cations per formula unit											
Si	0.995	0.981	0.991	0.979	0.985	0.982	0.986	0.978	0.981	0.991	0.988
Ti	0.000	0.000	0.000	0.000	0.000	0.000	0.000	0.001	0.000	0.000	0.000
Al	0.000	0.000	0.000	0.000	0.000	0.000	0.000	0.001	0.000	0.000	0.000
Cr	0.000	0.001	0.000	0.001	0.000	0.000	0.001	0.000	0.001	0.000	0.000
Fe^2+^	0.251	0.228	0.232	0.193	0.219	0.202	0.223	0.201	0.206	0.233	0.219
Fe^3+^	0.009	0.039	0.019	0.043	0.029	0.036	0.026	0.042	0.036	0.017	0.024
Mn	0.004	0.005	0.004	0.005	0.005	0.004	0.003	0.005	0.005	0.005	0.006
Ni	0.004	0.001	0.003	0.004	0.002	0.003	0.004	0.003	0.003	0.003	0.003
Mg	1.736	1.735	1.750	1.775	1.758	1.771	1.756	1.768	1.766	1.750	1.759
Ca	0.000	0.008	0.000	0.000	0.000	0.000	0.000	0.001	0.001	0.000	0.001
Na	0.000	0.002	0.000	0.001	0.000	0.001	0.000	0.001	0.000	0.000	0.000
K	0.000	0.000	0.000	0.000	0.001	0.000	0.000	0.000	0.000	0.000	0.000
Cations	3.000	3.000	3.000	3.000	3.000	3.000	3.000	3.000	3.000	3.000	3.000
Fo content	0.87	0.87	0.87	0.88	0.88	0.88	0.88	0.88	0.88	0.88	0.88

**Table 3:** Major elements composition (in wt. %) and atoms per formula unit of spinel from Premosello Al-rich diopside pyroxenites.

Sample ID	PR3-1	PR3-1	PR3-1	PR3-1	PR3-1	PR3-1	PR3-2	PR3-2	PR3-2	PR3-3	PR3-3	PR3-3	PR3-3
Crystal ID	25	26	27	28	29	30	31	32	33	34	35	36	37
Comments	Core	Rim	Core	Rim	Core	Rim	Core	Core	Core	Core	Core	Core	Core
SiO_2_	0.00	0.00	0.02	0.01	0.01	0.00	0.01	0.01	0.05	0.00	0.02	0.03	0.32
TiO_2_	0.00	0.01	0.00	0.01	0.04	0.00	0.06	0.00	0.00	0.00	0.00	0.00	0.00
Al_2_O_3_	66.67	65.60	66.90	66.91	66.52	66.62	67.01	66.76	65.96	66.13	66.93	66.62	62.89
Cr_2_O_3_	0.73	0.90	0.35	0.27	0.45	0.79	0.38	0.50	0.44	0.69	0.40	0.75	0.77
FeO_T_	11.88	12.04	11.80	11.75	11.72	11.11	13.02	13.02	13.76	12.31	11.99	12.09	11.96
MnO	0.11	0.15	0.11	0.08	0.15	0.07	0.13	0.16	0.18	0.07	0.15	0.15	0.13
NiO	0.42	0.37	0.35	0.39	0.43	0.40	0.14	0.22	0.08	0.30	0.26	0.31	0.25
MgO	20.96	21.41	21.02	20.64	20.88	21.12	19.77	20.21	20.48	21.00	20.89	20.38	20.32
CaO	0.00	0.00	0.00	0.02	0.01	0.03	0.00	0.00	0.00	0.00	0.03	0.02	0.02
Na_2_O	0.02	0.00	0.05	0.03	0.05	0.00	0.01	0.00	0.02	0.00	0.00	0.05	0.03
K_2_O	0.01	0.00	0.01	0.01	0.01	0.00	0.00	0.00	0.00	0.00	0.00	0.00	0.00
P_2_O_5_	0.00	0.04	0.00	0.02	0.00	0.00	0.00	0.02	0.00	0.01	0.01	0.02	0.00
Total	100.80	100.52	100.62	100.14	100.27	100.14	100.53	100.90	100.97	100.51	100.69	100.41	96.69
Normalization to 4 oxygens and 3 cations per formula unit													
Si	0.000	0.000	0.000	0.000	0.000	0.000	0.000	0.000	0.001	0.000	0.000	0.001	0.008
Ti	0.000	0.000	0.000	0.000	0.001	0.000	0.001	0.000	0.000	0.000	0.000	0.000	0.000
Al	1.951	1.926	1.958	1.969	1.955	1.958	1.976	1.961	1.937	1.942	1.960	1.961	1.922
Cr	0.014	0.018	0.007	0.005	0.009	0.016	0.008	0.010	0.009	0.014	0.008	0.015	0.016
Fe^2+^	0.211	0.195	0.208	0.219	0.208	0.205	0.258	0.242	0.234	0.213	0.218	0.228	0.212
Fe^3+^	0.036	0.056	0.037	0.026	0.037	0.027	0.014	0.029	0.053	0.044	0.031	0.025	0.048
Mn	0.002	0.003	0.002	0.002	0.003	0.002	0.003	0.003	0.004	0.002	0.003	0.003	0.003
Ni	0.008	0.007	0.007	0.008	0.009	0.008	0.003	0.004	0.002	0.006	0.005	0.006	0.005
Mg	0.776	0.795	0.778	0.768	0.776	0.785	0.737	0.750	0.760	0.780	0.773	0.758	0.785
Ca	0.000	0.000	0.000	0.001	0.000	0.001	0.000	0.000	0.000	0.000	0.001	0.001	0.001
Na	0.001	0.000	0.002	0.001	0.002	0.000	0.000	0.000	0.001	0.000	0.000	0.002	0.002
K	0.000	0.000	0.000	0.000	0.000	0.000	0.000	0.000	0.000	0.000	0.000	0.000	0.000
Cations	3.000	3.000	3.000	3.000	3.000	3.000	3.000	3.000	3.000	3.000	3.000	3.000	3.000
Mg#	0.76	0.76	0.76	0.76	0.76	0.77	0.73	0.73	0.73	0.75	0.76	0.75	0.75

**Table 4:** Major elements composition (in wt. %) and atoms per formula unit of amphibole from Premosello Al-rich diopside pyroxenites.

Sample ID	PR3-1	PR3-1	PR3-1	PR3-1	PR3-1	PR3-2	PR3-2	PR3-2	PR3-2	PR3-2	PR3-3	PR3-3	PR3-3	PR3-3	PR3-3
Crystal ID	38	39	40	41	42	43	44	45	46	47	48	49	50	51	52
Comments	Core	Core	Core	Core	Core	Core	Core	Core	Core	Core	Core	Core	Core	Core	Core
SiO_2_	44.02	42.86	43.17	42.50	43.91	42.17	42.51	42.37	42.72	42.83	42.46	42.16	42.44	42.91	42.82
TiO_2_	1.99	0.93	0.98	1.99	1.57	4.24	1.56	1.71	0.77	0.81	1.47	2.34	1.12	1.44	1.91
Al_2_O_3_	13.63	16.06	15.93	16.06	14.65	14.77	15.33	15.68	15.90	15.97	15.74	14.74	15.46	15.40	14.68
Cr_2_O_3_	0.03	0.18	0.12	0.10	0.03	0.02	0.10	0.07	0.05	0.08	0.10	0.04	0.16	0.13	0.05
FeO_T_	4.82	4.88	4.86	4.49	4.74	5.35	5.06	5.28	4.75	5.04	4.74	4.97	5.01	4.73	5.17
MnO	0.08	0.08	0.06	0.06	0.01	0.08	0.12	0.12	0.10	0.07	0.07	0.07	0.10	0.06	0.07
NiO	0.08	0.10	0.10	0.22	0.00	0.00	0.05	0.11	0.08	0.03	0.05	0.06	0.10	0.07	0.04
MgO	17.23	16.91	17.15	16.33	17.11	15.57	17.31	16.66	17.08	16.73	16.94	16.83	17.70	17.17	17.17
CaO	11.84	11.40	11.53	11.62	11.76	11.90	12.12	12.10	11.71	11.82	12.14	12.02	11.99	11.78	12.05
Na_2_O	3.74	3.70	3.50	3.74	3.64	3.49	3.75	3.73	3.71	3.65	3.82	3.76	3.76	3.43	3.77
K_2_O	0.43	0.49	0.40	0.32	0.14	0.18	0.01	0.00	0.53	0.52	0.04	0.13	0.36	0.51	0.17
P_2_O_5_	0.08	0.07	0.10	0.11	0.07	0.15	0.09	0.09	0.08	0.10	0.06	0.12	0.07	0.14	0.06
Cl	0.06	0.06	0.06	0.06	0.03	0.00	0.00	0.01	0.02	0.05	0.03	0.04	0.05	0.07	0.01
Total	98.03	97.72	97.96	97.61	97.67	97.91	98.00	97.94	97.49	97.71	97.66	97.29	98.31	97.84	97.97
Normalization to 23 oxygens and 16 cations per formula unit															
Si	6.266	6.113	6.135	6.068	6.242	6.031	6.059	6.048	6.110	6.120	6.062	6.065	6.043	6.120	6.110
Ti	0.213	0.100	0.104	0.214	0.167	0.456	0.167	0.184	0.083	0.088	0.158	0.253	0.120	0.155	0.205
Al	2.287	2.700	2.668	2.702	2.454	2.489	2.575	2.638	2.680	2.689	2.648	2.499	2.595	2.588	2.469
Cr	0.004	0.020	0.013	0.012	0.004	0.002	0.011	0.008	0.005	0.010	0.011	0.005	0.018	0.015	0.005
Fe^2+^	0.574	0.582	0.578	0.536	0.564	0.640	0.603	0.630	0.568	0.602	0.566	0.598	0.597	0.564	0.617
Fe^3+^	0.000	0.000	0.000	0.000	0.000	0.000	0.000	0.000	0.000	0.000	0.000	0.000	0.000	0.000	0.000
Mn	0.009	0.010	0.008	0.007	0.001	0.009	0.015	0.015	0.013	0.009	0.009	0.009	0.012	0.007	0.008
Ni	0.009	0.012	0.012	0.025	0.000	0.000	0.006	0.013	0.009	0.004	0.006	0.007	0.012	0.007	0.004
Mg	3.653	3.592	3.630	3.472	3.622	3.316	3.675	3.542	3.638	3.560	3.602	3.606	3.754	3.647	3.649
Ca	1.806	1.742	1.756	1.778	1.791	1.823	1.851	1.851	1.795	1.810	1.857	1.853	1.829	1.800	1.842
Na	1.032	1.023	0.964	1.035	1.003	0.968	1.036	1.032	1.029	1.011	1.057	1.049	1.038	0.948	1.043
K	0.077	0.089	0.072	0.059	0.026	0.033	0.001	0.000	0.096	0.095	0.007	0.024	0.065	0.093	0.031
Cations	15.93	15.98	15.94	15.91	15.88	15.77	16.00	15.96	16.03	16.00	15.98	15.97	16.08	15.94	15.98
Mg#	0.86	0.86	0.86	0.87	0.87	0.84	0.86	0.85	0.86	0.86	0.86	0.86	0.86	0.87	0.86

**Table 5:** Trace elements composition (in ppm) of clinopyroxene (crystal 1-13) and orthopyroxene (crystal 53-55) from Premosello Al-rich diopside pyroxenites.

Sample ID	PR3-1	PR3-1	PR3-1	PR3-1	PR3-1	PR3-2	PR3-2	PR3-2	PR3-2	PR3-3	PR3-3	PR3-3	PR3-3	PR3-2
Crystal ID	1	2	3	4	5	6	7	8	9	10	11	12	13	53-55
Comments	Core	Core	Core	Core	Core	Core	Core	Core	Core	Core	Core	Core	Core	*n* = 3
Li	1.03	1.47	1.28	1.01	1.76	1.78	1.47	1.29	1.13	1.12	1.68	1.12	1.26	0.60
Be	0.37	0.33	0.46	0.12	0.14	0.35	0.11	0.29	0.50	0.31	0.44	0.30	0.99	bdl
B	11.68	12.81	16.48	15.08	11.67	8.99	4.21	4.28	8.97	20.01	14.90	13.77	22.52	7.97
Sc	77.6	72.7	68.8	68.4	64.9	72.8	71.9	73.0	73.3	74.4	76.0	73.7	74.2	19.48
V	344	346	339	348	290	356	345	358	366	372	362	342	364	154
Cr	148	74	151	168	129	180	167	218	206	250	239	218	229	101
Mn	541	527	563	612	645	733	753	678	671	620	537	562	594	1809
Co	12.97	12.66	13.70	13.73	13.69	14.29	13.76	12.70	14.08	16.36	13.79	14.42	15.62	51.7
Ni	154	127	98.1	86.7	87.6	81.8	80.0	74.9	80.7	118	110	97.5	96.2	210
Cu	0.58	0.40	0.49	0.37	0.61	0.33	0.33	0.69	0.62	0.33	0.67	0.39	0.35	0.058
Zn	7.23	5.92	4.51	7.39	5.62	3.73	4.54	4.73	5.54	15.20	9.05	7.80	8.30	17.97
Rb	0.02	0.02	0.02	0.01	0.12	0.01	0.01	0.01	0.01	0.04	0.01	0.03	0.03	0.011
Sr	137.3	64.73	63.91	58.80	62.81	72.15	71.20	62.61	67.66	73.58	98.39	89.81	110.0	0.36
Y	17.66	25.01	25.71	27.51	21.40	29.51	29.55	30.08	24.15	30.89	29.58	26.76	26.92	1.12
Zr	29.13	34.11	32.38	33.30	27.65	37.17	38.01	34.55	35.38	38.89	39.08	34.50	36.11	1.47
Nb	bdl	0.003	0.024	0.017	0.016	0.014	0.009	0.028	bdl	0.018	0.015	0.028	0.025	0.002
Cs	0.01	0.01	0.01	0.01	0.01	0.01	0.01	0.01	0.01	0.02	0.02	0.02	0.01	0.012
Ba	0.82	0.09	0.95	0.09	1.28	0.10	0.08	0.10	0.14	0.10	0.43	0.30	0.95	0.033
La	5.18	4.44	4.32	4.88	4.42	5.85	5.71	5.13	4.57	5.30	4.93	5.07	4.84	0.017
Ce	16.08	19.84	19.04	20.71	17.54	24.18	23.24	21.60	18.47	22.46	19.76	19.69	18.57	0.082
Pr	2.84	3.99	3.80	3.97	3.58	4.83	4.53	4.22	3.69	4.66	3.72	3.92	3.72	0.014
Nd	16.34	23.67	23.18	24.98	21.13	27.58	27.95	27.25	22.87	29.07	24.74	25.89	23.04	0.112
Sm	5.21	7.25	8.00	8.94	7.24	8.31	8.69	8.90	7.75	10.52	9.68	7.46	9.09	0.028
Eu	1.67	1.99	2.27	2.09	1.91	2.60	2.49	2.38	2.18	2.48	2.09	2.12	2.08	0.022
Gd	5.10	7.39	7.46	7.73	6.28	8.41	8.66	8.69	6.88	9.08	8.45	7.56	7.32	0.035
Tb	0.70	1.06	1.12	1.09	0.91	1.36	1.17	1.22	1.13	1.39	1.23	1.06	1.17	0.020
Dy	3.94	5.42	5.85	6.07	5.06	6.61	6.78	6.80	5.63	6.84	6.73	6.35	5.57	0.148
Ho	0.65	0.89	1.04	1.16	0.89	1.24	1.19	1.19	1.02	1.27	1.38	1.13	1.00	0.049
Er	1.87	2.77	2.60	2.49	2.25	2.74	3.01	2.76	2.67	3.27	3.11	2.82	2.34	0.125
Tm	0.22	0.34	0.28	0.34	0.29	0.42	0.40	0.35	0.34	0.38	0.32	0.35	0.36	0.030
Yb	1.60	2.50	2.08	2.25	1.72	2.58	2.47	2.39	1.96	2.65	2.00	1.80	1.87	0.258
Lu	0.21	0.28	0.30	0.28	0.22	0.28	0.33	0.33	0.23	0.30	0.24	0.24	0.29	0.069
Hf	1.15	1.04	0.99	1.29	1.13	1.70	1.75	1.51	1.85	1.77	1.79	1.22	1.73	0.091
Ta	0.003	0.003	0.013	0.006	0.007	0.008	0.002	0.006	0.002	0.006	0.005	0.004	0.031	bdl
Pb	0.44	0.29	0.25	0.32	0.07	0.07	0.11	0.34	0.34	0.45	0.43	0.43	0.19	0.018
Th	0.34	0.32	0.19	0.20	0.16	0.20	0.14	0.25	0.22	0.21	0.28	0.32	0.29	0.005
U	0.087	0.104	0.037	0.041	0.023	0.012	0.009	0.036	0.085	0.076	0.051	0.073	0.033	0.002

bdl – below detection limit.

**Table 6:** Trace elements composition (in ppm) of olivine from Premosello Al-rich diopside pyroxenites*.

Sample ID	PR3-1	PR3-1	PR3-1	PR3-1	PR3-2	PR3-2	PR3-2	PR3-3	PR3-3	PR3-3	PR3-3
Crystal ID	14	15	16	17	18	19	20	21	22	23	24
Comments	Core	Core	Core	Core	Core	Core	Core	Core	Core	Core	Core
Li	2.74	3.93	5.15	1.57	3.12	4.33	3.22	2.21	3.12	2.27	1.52
Be	bdl	bdl	bdl	bdl	bdl	0.09	bdl	0.12	bdl	bdl	bdl
B	16.09	18.42	23.23	15.31	9.89	8.86	9.21	24.90	18.88	21.81	27.77
Sc	2.16	1.47	1.65	2.08	0.91	0.69	0.91	1.20	0.91	1.11	1.01
V	0.49	0.36	0.39	0.87	0.27	0.10	0.25	0.10	0.12	0.18	6.23
Cr	1.08	0.86	0.96	0.91	1.24	2.42	1.73	1.39	1.12	1.16	1.24
Mn	1719	1722	1826	1695	1784	1957	2094	1816	1910	1762	1907
Co	139	128	151	138	174	165	128	127	159	142	137
Ni	1257	1198	1253	1245	1441	1242	1067	1353	1328	1313	1282
Cu	0.07	0.07	0.07	0.05	0.07	0.05	0.06	0.10	0.09	0.09	0.12
Zn	16.47	14.84	25.47	18.55	47.54	41.21	17.35	19.03	36.79	22.76	21.16
Rb	0.02	0.02	0.02	0.02	0.02	0.02	0.02	0.03	0.04	0.02	0.04
Sr	0.02	0.01	0.01	0.00	0.04	0.06	0.01	0.02	0.03	0.03	0.04
Y	0.012	0.008	0.093	0.031	0.031	0.028	0.011	0.014	0.014	0.007	0.015
Zr	0.022	0.012	0.006	0.060	0.007	0.006	0.028	0.019	0.022	bdl	0.031
Nb	0.004	bdl	bdl	bdl	0.006	0.007	bdl	0.005	bdl	bdl	bdl
Cs	0.010	0.011	0.009	0.013	0.012	0.009	0.013	0.019	0.018	0.021	0.031
Ba	0.013	0.000	0.014	0.006	0.009	0.019	0.010	0.025	0.011	0.006	0.025
Pb	0.046	0.023	0.067	0.049	0.027	0.021	0.041	0.069	0.055	bdl	0.079

bdl – below detection limit.

⁎ REEs, Hf, Ta, Th and U in analysed olivine are below detection limit (bdl).

**Table 7:** Trace elements composition (in ppm) of spinel from Premosello Al-rich diopside pyroxenites*.

Sample ID	PR3-1	PR3-1	PR3-1	PR3-1	PR3-1	PR3-1	PR3-2	PR3-2	PR3-2	PR3-3	PR3-3	PR3-3	PR3-3
Crystal ID	25	26	27	28	29	30	31	32	33	34	35	36	37
Comments	Core	Rim	Core	Rim	Core	Rim	Core	Core	Core	Core	Core	Rim	Core
Li	1.55	0.93	0.85	1.01	0.81	1.23	3.56	1.79	1.05	1.45	1.61	2.06	1.81
Be	0.51	0.71	0.18	0.40	0.35	0.59	bdl	bdl	bdl	bdl	0.24	0.42	0.11
B	36.31	32.27	29.65	36.25	32.65	29.53	11.63	5.92	10.81	31.82	27.44	38.62	26.63
Sc	0.13	0.13	0.17	0.12	0.10	0.11	0.20	0.07	0.09	0.17	0.18	0.20	0.17
V	296	307	296	279	270	287	586	354	484	296	276	304	222
Cr	5302	5757	2360	2629	2710	3066	2608	2931	3022	3397	4952	4470	4484
Mn	891	870	876	848	868	875	1008	1003	1094	958	963	909	863
Co	298	300	309	312	281	288	342	290	286	323	389	333	340
Ni	3722	3221	3115	2886	3419	3382	1278	1434	1480	1936	1985	2010	1942
Cu	0.50	0.08	0.06	9.33	0.06	0.15	0.10	0.05	0.05	0.12	0.12	0.15	1.43
Zn	2008	2055	1890	1912	1865	1916	1129	1251	1016	1527	2013	2292	3316
Rb	0.03	0.02	0.02	0.02	0.02	0.02	0.01	0.02	0.02	0.03	0.04	0.06	0.05
Sr	0.06	0.06	0.05	0.20	0.06	0.08	0.52	0.01	0.12	0.49	bdl	0.11	0.15
Y	0.009	0.000	0.007	0.008	0.007	0.002	0.016	0.005	0.006	0.015	bdl	0.015	0.025
Zr	0.017	0.041	bdl	0.014	0.005	0.005	0.137	0.032	bdl	0.038	0.023	0.052	0.027
Nb	0.010	0.019	bdl	0.006	0.007	0.003	0.005	0.003	0.011	0.005	0.000	0.006	0.015
Cs	0.015	0.012	0.010	0.013	0.013	0.008	0.012	0.014	0.008	0.020	0.035	0.043	0.045
Ba	0.050	0.103	0.011	0.371	0.113	0.096	1.049	0.019	0.152	0.163	0.054	0.405	0.117
Pb	0.054	0.029	0.060	0.065	0.033	0.042	0.033	0.046	0.033	0.079	0.058	bdl	0.098

bdl – below detection limit.

⁎ REEs, Hf, Ta, Th and U in analysed spinel are below detection limit (bdl).

**Table 8:** Trace elements composition (in ppm) of amphibole from Premosello Al-rich diopside pyroxenites.

Sample ID	PR3-1	PR3-1	PR3-1	PR3-1	PR3-1	PR3-2	PR3-2	PR3-2	PR3-2	PR3-2	PR3-3	PR3-3	PR3-3	PR3-3	PR3-3
Crystal ID	38	39	40	41	42	43	44	45	46	47	48	49	50	51	52
Comments	Core	Core	Rim	Rim	Core	Core	Core	Core	Core	Core	Core	Core	Core	Core	Core
Li	0.81	1.63	1.69	0.58	1.01	0.57	0.58	0.76	0.72	1.06	1.02	1.13	1.08	1.15	1.06
Be	1.21	0.81	0.57	0.48	0.79	bdl	0.16	0.11	0.75	0.95	0.64	0.64	0.61	0.70	0.90
B	25.55	26.58	22.91	21.39	17.91	10.43	10.75	6.38	12.04	7.73	28.16	18.85	17.66	22.74	17.33
Sc	59.6	52.5	57.3	69.2	58.8	75.0	56.3	57.0	55.5	54.2	57.9	64.4	59.2	61.4	60.0
V	477	503	544	532	477	691	646	680	375	375	515	528	426	410	506
Cr	216	798	430	249	520	321	371	442	668	724	410	360	466	391	381
Mn	531	585	519	496	494	607	643	656	540	554	509	491	472	482	494
Co	32.96	32.19	32.45	31.38	32.51	32.82	28.98	28.58	35.59	36.82	31.54	34.95	38.79	35.70	34.88
Ni	628	699	620	605	541	271	249	260	296	289	345	362	379	323	327
Cu	29.68	2.40	1.35	1.34	1.54	1.89	1.66	2.22	1.78	1.62	3.45	1.59	1.28	1.29	1.23
Zn	20.29	36.90	22.54	10.75	13.14	6.33	14.62	11.76	16.16	18.42	11.86	12.09	84.57	17.38	10.71
Rb	5.07	8.49	6.14	4.05	2.60	3.08	0.12	0.03	5.18	4.85	0.09	2.76	2.93	4.56	2.20
Sr	404	397	381	360	351	576	144	161	345	350	165	338	312	407	339
Y	25.46	24.31	20.28	23.33	27.69	64.57	32.48	34.71	24.57	23.86	32.13	44.04	25.69	27.23	34.17
Zr	28.25	22.65	24.63	32.08	27.20	33.36	25.83	27.03	26.02	25.03	30.56	32.45	30.35	30.21	28.67
Nb	1.83	3.28	0.10	0.13	0.18	1.64	0.12	0.16	5.77	3.32	0.05	0.93	0.03	0.59	0.57
Cs	0.051	0.090	0.069	0.032	0.070	0.286	0.009	0.036	0.014	0.014	0.018	0.169	0.024	0.042	0.026
Ba	274	467	418	366	283	32.88	4.85	2.08	267	211	7.53	57.18	147	191	94.8
La	10.13	10.15	9.34	9.83	9.25	9.79	6.36	6.82	8.48	8.18	6.48	7.37	7.44	9.10	7.42
Ce	30.67	30.21	27.51	27.36	28.56	38.40	25.38	26.65	27.73	26.95	23.98	26.47	21.29	26.01	23.87
Pr	4.65	4.92	4.07	4.31	4.65	7.62	4.87	5.12	4.63	4.59	4.53	5.35	3.78	4.32	4.25
Nd	25.44	25.91	21.72	22.56	24.71	47.76	28.66	31.68	23.76	23.63	27.97	35.53	21.18	25.29	26.61
Sm	7.78	7.01	6.12	6.81	7.58	16.05	10.08	10.23	6.98	7.26	9.94	11.60	6.84	7.25	10.94
Eu	2.47	1.99	2.03	1.87	2.22	4.83	2.80	3.03	2.24	2.01	2.43	2.85	1.64	2.04	2.66
Gd	6.95	6.03	5.01	6.32	6.78	16.63	8.47	9.19	6.95	6.72	8.51	12.07	7.41	7.13	9.92
Tb	0.96	0.95	0.80	0.96	1.08	2.60	1.42	1.35	0.99	0.92	1.19	1.80	1.14	1.10	1.59
Dy	5.48	4.93	4.10	4.95	6.04	13.57	6.63	6.88	5.42	5.43	7.13	9.84	6.55	5.56	6.85
Ho	0.99	0.97	0.81	1.01	1.08	2.62	1.53	1.42	1.02	1.00	1.36	1.93	1.10	1.11	1.22
Er	2.58	2.34	2.10	2.24	2.75	6.42	3.38	3.14	2.39	2.35	3.62	4.25	2.56	2.36	3.45
Tm	0.28	0.31	0.25	0.32	0.36	0.79	0.43	0.54	0.36	0.35	0.32	0.53	0.29	0.31	0.43
Yb	2.45	2.09	1.83	2.05	2.68	5.02	2.92	3.23	2.65	2.32	2.66	2.99	2.06	2.50	2.74
Lu	0.24	0.28	0.21	0.27	0.32	0.72	0.39	0.48	0.25	0.29	0.41	0.40	0.25	0.30	0.33
Hf	1.21	0.91	0.99	1.01	1.08	1.46	0.97	1.18	1.13	1.13	1.29	1.60	1.45	1.34	1.31
Ta	0.042	0.011	0.019	0.006	0.019	0.087	bdl	0.021	0.002	bdl	0.010	0.074	0.005	0.047	0.049
Pb	1.49	1.46	1.23	1.54	1.42	0.91	1.04	0.92	1.27	1.22	0.99	1.50	1.32	1.30	1.29
Th	0.43	0.47	0.41	0.35	0.42	0.17	0.20	0.23	0.37	0.42	0.20	0.29	0.22	0.40	0.38
U	0.030	0.113	0.172	0.106	0.175	0.008	0.054	0.026	0.096	0.124	0.069	0.067	0.064	0.051	0.073

Rare earth elements (REE) patterns of clinopyroxene and amphibole (Fig. 4a) from the studied pyroxenites are similar, exhibiting strong fractionation and distinct enrichments in LREEs and MREEs over the HREEs. For comparison, the compositions of clinopyroxene and amphibole in the studied pyroxenite are plotted alongside the compositions of clinopyroxenes from (i) spinel lherzolite and Al-augite pyroxenite [2,6] from the Premosello peridotite massif (Fig. 4a), and (ii) Cr-diopside and Al-augite pyroxenites [3,8] from the Balmuccia peridotite massif (Fig. 4b).

## 4 Experimental Design, Materials and Methods

### 4.1 Materials and methods

Mineral assemblages and textural features of the Premosello Al-rich diopside pyroxenite samples were determined by polarized light optical microscopy on thin sections at the Department of Earth and Environmental Sciences, University of Pavia (Italy).

#### 4.1.1 Electron microprobe and LA-ICP-MS analysis

The major element composition of mineral phases (clinopyroxene, olivine, spinel and amphibole) in samples of the Premosello Al-rich diopside pyroxenite were measured on thin sections by electron microprobe analysis (EMPA) using a JEOL JXA-8230 Superprobe equipped with five WDS spectrometers operating in wavelength dispersive mode, housed at the Joint Laboratory of the Department of Earth Sciences, University of Florence and the CNR-IGG Florence (Italy). Operating conditions were 15 kV accelerating voltage, 20 nA beam current, 3 µm spot size, and a counting time of 15 s on the peaks and 7 s on the backgrounds. Natural minerals (olivine for Mg; albite for Si and Na; ilmenite for Fe and Ti; bustamite for Mn; sanidine for K; plagioclase for Al; diopside for Ca; metallic nickel for Ni; chromite for Cr) were used as standards. The analytical results were corrected for matrix effects using the conventional ZAF method provided by the JEOL software package. Results are accurate and precise within 2–5%, as estimated by analysis of natural mineral standards performed during each analytical session.

Trace element contents of mineral phases (clinopyroxene, olivine, spinel and amphibole) were measured on thin sections using an Agilent 8900 QQQ-ICP-MS coupled to a 266 nm Nd:YAG laser ablation system at the CNR-IGG Pavia (Italy). The ICP-MS was tuned using NIST SRM 610 synthetic glass to optimize the signal intensity and stability by monitoring ^24^Mg, ^115^In, ^232^Th, and ^238^U; to check for mass bias using the ^232^Th/^238^U ratio; and to monitor the level of oxide formation in the plasma using the ^232^Th/^248^ThO ratio. The laser was operated at a repetition rate of 10 Hz, fluence of 8 J/cm^2^ and 50 µm spot size. Total acquisition time was 150 s per analysis, allowing about 50 s for background followed by 60 s for laser ablation. The following isotopes of major elements were also analysed: ^23^Na, ^25^Mg, ^27^Al, ^29^Si, ^39^K, ^43^Ca, ^44^Ca, ^49^Ti and ^57^Fe. Results obtained for the two isotopes analysed for Ca do not display any systematic differences, showing that no mass interferences occurred during analytical runs. NIST SRM 610 was used as an external standard. ^44^Ca was used as internal standard for clinopyroxene and amphibole; ^29^Si for olivine and orthopyroxene; and ^27^Al for spinel. Data reduction was done with the GLITTER software [19]. USGS reference sample BCR-2 [20] was repeatedly analysed together with the unknowns to assess precision and accuracy during the analytical sessions. Both statistical parameters were within 0 to 10% of the accepted standard concentration values [20] for most analysed elements, except for Cu, Zn, Nb and Ta (see full dataset in Ogunyele et al. [1]).

## Limitations

None.

## Ethics Statement

The authors declare that they have read and follow the ethical requirements for publication in Data in Brief and confirm that the current work does not involve human subjects, animal experiments, or any data collected from social media platforms.

## Credit author statement

**Abimbola C. Ogunyele:** Conceptualization, Methodology, Investigation, Formal Analysis, Writing – original draft, Writing – review & editing. **Alessio Sanfilippo:** Conceptualization, Investigation, Funding acquisition, Supervision, Writing – review & editing. **Mattia Bonazzi:** Investigation, Formal Analysis, Writing – review & editing. **Maria C. Lopez Suarez:** Investigation, Writing – review & editing. **Alberto Zanetti:** Conceptualization, Methodology, Investigation, Validation, Funding acquisition, Supervision, Writing – review & editing.

## Data Availability

Mendeley DataEMPA & LA-ICP-MS data of Al-diopside pyroxenites from Premosello peridotite (Original data).
